# Dexamethasone in Patients Hospitalized with COVID-19: Whether, When and to Whom

**DOI:** 10.3390/jcm10081607

**Published:** 2021-04-10

**Authors:** Luigino Calzetta, Marina Aiello, Annalisa Frizzelli, Paola Rogliani, Alfredo Chetta

**Affiliations:** 1 Respiratory Disease and Lung Function Unit, Department of Medicine and Surgery, University of Parma, 43125 Parma, Italy; marina.aiello@unipr.it (M.A.); annalisa.frizzelli@unipr.it (A.F.); alfredoantonio.chetta@unipr.it (A.C.); 2 Unit of Respiratory Medicine, Department of Experimental Medicine, University of Rome “Tor Vergata”, 00133 Rome, Italy; paola.rogliani@uniroma2.it

**Keywords:** dexamethasone, clinical interpretation, COVID-19, mortality, NNH, NNT

## Abstract

A clinical interpretation of the Randomized Evaluation of COVID-19 Therapy (RECOVERY) study was performed to provide a useful tool to understand whether, when, and to whom dexamethasone should be administered during hospitalization for COVID-19. A post hoc analysis of data published in the preliminary report of the RECOVERY study was performed to calculate the person-based number needed to treat (NNT) and number needed to harm (NNH) of 6 mg dexamethasone once daily for up to 10 days vs. usual care with respect to mortality. At day 28, the NNT of dexamethasone vs. usual care was 36.0 (95%CI 24.9–65.1, *p* < 0.05) in all patients, 8.3 (95%CI 6.0–13.1, *p* < 0.05) in patients receiving invasive mechanical ventilation, and 34.6 (95%CI 22.1–79.0, *p* < 0.05) in patients receiving oxygen only (with or without noninvasive ventilation). Dexamethasone increased mortality compared with usual care in patients not requiring oxygen supplementation, leading to a NNH value of 26.7 (95%CI 18.1–50.9, *p* < 0.05). NNT of dexamethasone vs. usual care was 17.3 (95%CI 14.9–20.6) in subjects <70 years, 27.0 (95%CI 18.5–49.8) in men, and 16.2 (95%CI 13.2–20.8) in patients in which the onset of symptoms was >7 days. Dexamethasone is effective in male subjects < 70 years that require invasive mechanical ventilation experiencing symptoms from >7 days and those patients receiving oxygen without invasive mechanical ventilation; it should be avoided in patients not requiring respiratory support.

## 1. Introduction

The updated guidance of the American Thoracic Society (ATS) and European Respiratory Society (ERS) International Task Force for the management of coronavirus disease 2019 (COVID-19) has recently suggested to use dexamethasone in hospitalized patients who require supplemental oxygen or are mechanically ventilated [1]. Such a recommendation arises from the evidence provided by the Randomized Evaluation of COVID-19 Therapy (RECOVERY) study [2], a multi-center, open-label, randomized trial that assigned 6 mg dexamethasone to patients hospitalized with COVID-19, taken once daily for up to 10 days, or usual care.

Despite the robustness of data provided by the RECOVERY study [2], results expressed as Kaplan–Meier survival curves and mortality rate ratios remain difficult to interpret, from a strictly clinical viewpoint, for those clinicians that are not accustomed to statistics. 

In this respect, the analysis of the number needed to treat (NNT) and number needed to harm (NNH) represents another way to assess the benefit and harm of a given therapeutic option, a validated approach to evaluate the clinical impact of corticosteroids in respiratory disorders [3,4]. Well-performed NNT and NNH analyses have the advantage of providing suitable information on the real absolute sizes of treatment effects, and when 95% confidence interval (95%CI) values are also reported, they convey both clinical as well as statistical significance [5].

Therefore, a careful interpretation of data reported in the supplementary appendix and preliminary report of RECOVERY study [2] via the analysis of NNT and NNH may provide a useful tool for clinical decision making to understand whether, when, and to whom dexamethasone should be administered during hospitalization for COVID-19.

## 2. Materials and Methods

A post hoc analysis was performed to calculate the values of NNT and NNH, representing the reciprocal of the absolute risk reduction (ARR) or increase (ARI), respectively, associated with an intervention over a fixed period of time [5,6,7].

The main outcome of the RECOVERY study [2] was all-cause mortality reported via rate ratio, a relative measure of mortality. According to the primary publication [2], the outcome of this post hoc analysis was always the always the all-cause mortality, but reported as NNT and NNH that are absolute measures of mortality. Specifically, NNT referred to a reduction in mortality (benefit) and NNH referred to an increase in mortality (harm).

In this study, the values of NNT and NNH are reported as person-based and calculated by analyzing the Kaplan–Meier survival curves or by using raw mortality data for dexamethasone and usual care provided in the primary publication [2], as previously described [8,9]. The NNT and NNH with 95%CI are the reciprocal of the ARR or ARI in mortality, respectively, associated with dexamethasone vs. usual care over 7, 14, 21, and 28 days since randomization. 

NNT and NNH were calculated as follows: NNT = 1/ARR and NNH = 1/AAI, respectively; the 95%CI of NNT and NNH were calculated as follows: 95%CI of NNT = ARR ± 1.96 * SE(ARR) and NNH = ARR ± 1.96 * SE(ARI), where SE is the standard error [8,9]. ARR or ARI are the arithmetic differences between two event rates and were calculated as follows: ARR or ARI = ARC − ART, where ARC and ART are the absolute risk (AR) in the control and treatment groups, respectively; AR is the number of events in control and treatment groups, divided by the number of patients in that group [8,9]. The SE of ARR or ARI were calculated according with the equation proposed by Altman and Andersed [8] as follows: SE(ARR) or SE (ARI) = √[S_a_^2^(1 − S_a_)/n_a_ + S_c_^2^(1 − S_c_)/n_c_], where S_a_ and S_c_ are the survival probabilities in the dexamethasone and usual care groups, respectively, n_a_ and n_c_ are the number of patients still at risk (alive) in the dexamethasone and usual care groups, respectively.

Subset analyses were carried out on age, sex, and onset of symptoms to identify potentially confounding factors with respect to the NNT and NNH of dexamethasone vs. usual care.

ImageJ was used to extract data from the figures, when necessary [10] and GraphPad Prism (CA, US) software to graph the data. The statistical significance of the effect estimates resulting from the network meta-analysis was assessed for *p* < 0.05.

## 3. Results

### 3.1. Extracted Data

Details regarding the raw data extracted at specific time-points from the RECOVERY study [2] according with treatment and respiratory support in patients hospitalized with COVID-19 are reported in Table 1.

### 3.2. NNT

**Figure 1:**
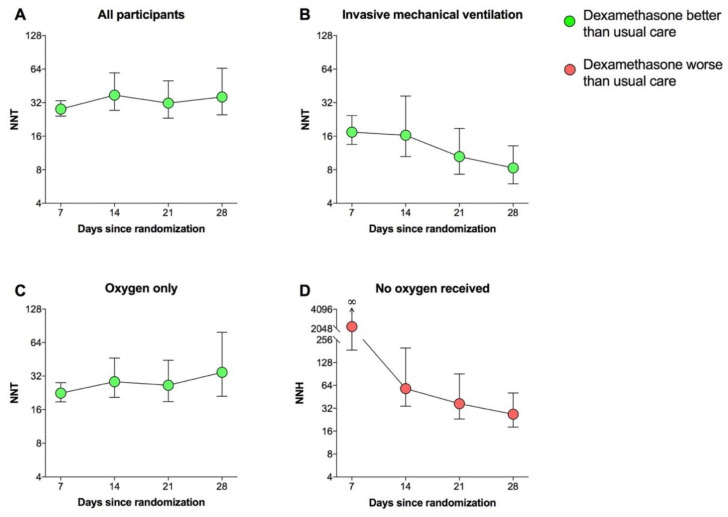
Graphical representation (**A**–**D**) of NNT and NNH with 95%CI of dexamethasone vs. usual care on mortality at 7, 14, 21, and 28 days according to respiratory support as reported by the RECOVERY study in patients hospitalized with COVID-19. ∞: infinity; CI: confidence interval; NNH: number needed to harm; NNT: number need to treat.

The overall NNT of dexamethasone vs. usual care was significant (*p* < 0.05) and ranged between 28.0 and 37.4 (Figure 1A). The subgroup analysis in patients receiving invasive mechanical ventilation showed a significant (*p* < 0.05) and time-dependent trend for which the NNT values of dexamethasone vs. usual care started from 17.4 at 7 days since randomization and improved up to 8.5 after four weeks of treatment (Figure 1B). Among patients receiving oxygen without invasive mechanical ventilation, the treatment with dexamethasone was significantly (*p* <0.05) superior to usual care, with NNT values ranging between 22.5 and 32.0 (Figure 1C).

Details on NNT are shown in Table 2 along with 95%CI values and statistical significance.

### 3.3. NNH

While the RECOVERY study [2] reported no clear effect of dexamethasone vs. usual care among patients who were not receiving any respiratory support at randomization (rate ratio: 1.19, 95%CI 0.91–1.55, *p* > 0.05), our analysis shows an evident and statistically significant (*p* < 0.05) harm of dexamethasone vs. usual care after 14 days since randomization, with the worst NNH value of 26.7 at the end of the study (Figure 1D). Details on NNH are shown in Table 2 along with 95%CI values and statistical significance.

### 3.4. Subset Analyses

When considering potentially confounding factors according with the primary publication [2], we found significant (*p* < 0.05) NNT values of dexamethasone vs. usual care in subjects younger than 70 years (17.3 95%CI 14.9–20.6), in men (27.0 95%CI 18.5–49.8), and in those patients in which the onset of symptoms was >7 days (16.2 95%CI 13.2–20.8). In subjects ≥70 years of age, women, and those patients in which the onset of symptoms was ≤7 days, no significant (*p* > 0.05) NNT values were detected (35.4 95%CI 13.7–∞, 86.8 95%CI 34.6–∞, 63.3 95%CI 25.7–∞, respectively).

## 4. Discussion

The results of this analysis provide the evidence that only ≈8 patients receiving invasive mechanical ventilation had to be treated with dexamethasone to prevent one death compared to usual care over 4 weeks, whereas in patients receiving oxygen only (with or without noninvasive ventilation), one death was prevented for every 35 patients. Thus, it seems that the benefits of dexamethasone are related with the severity of illness, at least in those patients receiving respiratory support. In fact, in less severe patients not requiring oxygen supplementation, dexamethasone not only provided no benefit but it even induced a significant harm, leading to one death every ≈27 patients when compared with usual care.

Interestingly, our analysis supports the possibility that dexamethasone may induce harm in patients who did not require oxygen as resulting from the RECOVERY study [2]. In this regard, we found that the risk of death expressed as NNH was statistically significant for dexamethasone vs. usual care despite in the primary publication [2] there was only a trend toward harm that, however, was not significant when considering the rate ratio. Indeed, such an inconsistency on the level of significance is worthy of consideration. First, the P-value does not inform us as to whether the results can be clinically significant or how often an event can be encountered in clinical practice [11]. Second, the NNH resulting in our analysis and the rate ratio reported in the RECOVERY study [2] express the same outcome via different metrics, namely the absolute and relative measures of harm, respectively, thus explaining the possibility of different levels of P-values [11,12]. Third, both NNT and NNH can be independent of the P-value, and it is possible to calculate them even from not statistically significant relative measures [12]. Therefore, the results of this analysis should be interpreted free from the “tyranny of the P” that leads researchers astray and confuses clinicians eager for guidance as to the benefit/harm of the investigated intervention [12]. Certainly, both NNT and NNH remain suitable tools of effect size measures in helping to interpret the results of clinical trial and allowing clinicians to evaluate the differences between specific interventions [11,12]. Ultimately, to the best of our knowledge, our study provides, for the first time, a proper analysis of the NNT and NNH of dexamethasone in patients hospitalized with COVID-19.

Therefore, also in the light of the NNT values resulting from potentially confounding factors, the hospitalized COVID-19 patients that may receive the greatest benefit from the treatment with 6 mg dexamethasone once daily for up to 10 days are those male subjects younger than 70 years that require invasive mechanical ventilation and experiencing symptoms from more than 7 days, followed by those receiving oxygen without invasive mechanical ventilation. While in women and subjects elderly than 70 years dexamethasone seems to induce neither benefit nor harm, the findings provided by our analysis indicate that its use should be avoided in those patients not requiring respiratory support.

The reasons why dexamethasone is more effective in patients receiving respiratory support and in those enrolled after the first week of disease have already been discussed in the preliminary report of RECOVERY study [2], whereas the lack of effectiveness in women has not yet been addressed. Perhaps, this lack of response to dexamethasone may be related with the intrinsic estrogen/progesterone receptors expression in women who are already protected from SARS-CoV-2 infection due to circulating sex steroids, as already demonstrated [13,14].

Although it has been suggested that the treatment beneficial effect on mortality is a general effect of class of corticosteroids and not specific to any particular agent [15], solid evidence is currently available only for dexamethasone. Indeed, further large and well-designed trials are needed to assess whether other corticosteroids may have the same benefit-harm profile of dexamethasone. Finally, there is also the medical need of specific studies to assess whether, and eventually why, corticosteroids may increase the risk of mortality in hospitalized COVID-19 patients not requiring respiratory support, although the evidence of our analysis raises potential ethical concerns of performing such investigations.

## Figures and Tables

**Table 1:** Raw data concerning alive and dead patients extracted from the RECOVERY study at 7, 14, 21, and 28 days according with treatment and respiratory support in patients hospitalized with COVID-19.

Days since Randomization	Treatment	All Participants		Invasive Mechanical Ventilation		Oxygen Only		No Oxygen Received	
		Alive	Dead	Alive	Dead	Alive	Dead	Alive	Dead
7	Dexamethasone	1903	201	290	34	1135	144	478	23
	Usual care	3754	567	572	111	2195	409	987	47
14	Dexamethasone	1725	379	248	76	1036	243	441	60
	Usual care	3427	894	481	202	2018	586	928	106
21	Dexamethasone	1659	445	232	92	1006	273	421	80
	Usual care	3271	1050	424	259	1950	654	897	137
28	Dexamethasone	1622	482	229	95	981	298	412	89
	Usual care	3211	1110	400	283	1922	682	889	145

**Table 2:** NNT and NNH with 95%CI values of dexamethasone vs. usual care on mortality at 7, 14, 21, and 28 days according to respiratory support as reported by the RECOVERY study in patients hospitalized with COVID-19. NNT values indicate that dexamethasone was better than usual care, whereas significant NNH values imply that dexamethasone was worse than usual care.

Days Since Randomization	NNT			NNH
	All Participants	Invasive Mechanical Ventilation	Oxygen Only	No Oxygen Received
7	28.0 (24.2–33.3) *	17.4 (24.5–13.5) *	22.5 (18.8–27.9) *	2204.4 (187.4–∞)
14	37.4 (27.3–59.2) *	16.3 (10.5–36.6) *	28.5 (20.6–46.5) *	58.0 (34,198.5) *
21	31.7 (23.2–50.2) *	10.5 (7.3–18.8) *	26.5 (18.9–44.4) *	36.8 (23.1–90.6) *
28	36.0 (24.9–65.1) *	8.3 (6.0–13.1) *	34.6 (21.1–79.0) *	26.7 (18.1–50.9) *

* *p* < 0.05 of dexamethasone vs. usual care. ∞: infinity; CI: confidence interval; NNH: Number needed to harm; NNT: Number need to treat.

## Data Availability

The datasets generated during the current study are available from the corresponding author on reasonable request.
